# Antioxidative, Anti-Inflammatory, and Anticancer Effects of Purified Flavonol Glycosides and Aglycones in Green Tea

**DOI:** 10.3390/antiox8080278

**Published:** 2019-08-05

**Authors:** Chan-Su Rha, Hyun Woo Jeong, Saitbyul Park, Siyoung Lee, Young Sung Jung, Dae-Ok Kim

**Affiliations:** 1 Vitalbeautie Research Division, Amorepacific Corporation R&D Center, Yongin 17074, Korea; 2 Safety and Regulatory Division, Amorepacific Corporation R&D Center, Yongin 17074, Korea; 3 Precision Medicine Research Center, Advanced Institutes of Convergence Technology, Suwon 16229, Korea; 4 Department of Food Science and Biotechnology, Kyung Hee University, Yongin 17104, Korea

**Keywords:** anticancer effect, anti-inflammatory effect, antioxidative effect, flavonol aglycone, flavonol glycoside, green tea

## Abstract

(1) Background: Extensive research has focused on flavan-3-ols, but information about the bioactivities of green tea flavonols is limited. (2) Methods: In this study, we investigated the antioxidative, anti-inflammatory, and anticancer effects of flavonol glycosides and aglycones from green tea using in vitro cell models. The fractions rich in flavonol glycoside (FLG) and flavonol aglycone (FLA) were obtained from green tea extract after treatment with tannase and cellulase, respectively. (3) Results: FLG and FLA contained 16 and 13 derivatives, respectively, including apigenin, kaempferol, myricetin, and quercetin, determined by mass spectrometry. FLA exhibited higher radical-scavenging activity than that of FLG. FLG and FLA attenuated the levels of intracellular oxidative stress in neuron-like PC-12 cells. The treatment of RAW 264.7 murine macrophages with FLG and FLA significantly reduced the mRNA expression of inflammation-related genes in a dose-dependent manner. Furthermore, FLG and FLA treatments decreased the viability of the colon adenoma cell line DLD-1 and breast cancer cell line E0771. Moreover, the treatment with FLG or FLA combined with paclitaxel had synergistic anticancer effects on the DLD-1 cell line. (4) Conclusions: Flavonols from green tea exerted beneficial effects on health and may be superior to flavan-3-ols.

## 1. Introduction

Green tea is a familiar beverage with high amounts of bioactive polyphenols, such as catechins (flavan-3-ols), flavones, and flavonols [1]. Green tea generally contains approximately 30% phenolic compounds, of which catechins and flavonols account for approximately 15% and 0.4% of the dry weight (DW), respectively [2,3]. Flavonols and flavones in green tea include myricetin, quercetin, apigenin, and kaempferol [3]. These green tea flavonols and flavones are mainly in the form of glycosides, and their content and compositions vary among tea cultivars [4,5].

Flavonols exhibit anticancer activity by inhibiting the proliferation and angiogenesis of cancer cells and exerting pro-apoptotic effects [6]. These anticancer effects have been demonstrated in several cancer cell lines, in addition to antioxidative and antihyperlipidemic effects [6,7,8]. The consumption of green tea has been reported to increase the plasma antioxidant capacity [9]. The effects of flavonol supplementation on cardiometabolic risk factors revealed significant reductions in triacylglycerol, total cholesterol, low-density lipoprotein, and fasting plasma glucose levels, as well as blood pressure, and a significant increase in high-density lipoprotein [10]. The antioxidative effects of flavonol glycosides are weaker than those of flavonol aglycones [11]. Several studies have suggested that green tea promotes physiological functions, including body weight regulation, ultraviolet protection, bone mineral density maintenance, and antibacterial, antihypertensive, antifibrotic, and neuroprotective activities [12,13].

Many health problems are strongly related to the inflammation status in humans [14]. The effects of green tea and its catechins to relieve inflammation in cells have been investigated [15,16]. Approximately 65% of adults exhibit downregulated intestinal lactase production [17]. Lactase or lactase-phlorizin hydrolase exists on the brush border membrane of enterocytes. Lactase catalyzes the hydrolysis of a variety of β-glucosides, including flavonoid glucosides [18]. Glycosylated flavonols and flavones have different sugar bonds and compositions depending on the plant source and are therefore subjected to different digestion processes in the body.

Studies on the diverse health-promoting effects of green tea have typically focused on catechins [12,13]. Unlike catechins, green tea flavonols have received little attention with respect to their usage and functionality. In this study, we investigated the antioxidative, anti-inflammatory, and anticancer effects of the fractions rich in flavonol glycoside (FLG) and flavonol aglycone (FLA) isolated from enzymatically treated green tea extract (GTE).

## 2. Materials and Methods

### 2.1. Chemicals, Reagents, and Cell Lines

Catechin, gallic acid, dimethyl sulfoxide (DMSO), aluminum chloride, ascorbic acid, Folin–Ciocalteu’s phenol reagent, 2,2′-azino-bis(3-ethylbenzothiazoline-6-sulfonic acid) diammonium salt (ABTS), 1,1-diphenyl-2-picrylhydrazyl (DPPH), 3-(4,5-dimethylthiazol-2yl)-2,5-diphenyltetrazolium bromide (MTT), H_2_O_2_, an in vitro lactate dehydrogenase toxicology assay kit, phosphate-buffered saline (PBS), lipopolysaccharide (LPS), penicillin/streptomycin, Roswell Park Memorial Institute (RPMI) 1640, Dulbecco’s modified Eagle’s medium (DMEM), 2′,7′-dichlorofluorescein diacetate (DCFH-DA), quercetin, apigenin, kaempferol, formic acid, and paclitaxel (code: Y0000698) were purchased from Sigma-Aldrich Co., LLC (St. Louis, MO, USA). Myricetin was purchased from Extrasynthese (Genay, France). 2,2′-Azobis(2-amidinopropane) dihydrochloride (AAPH), (−)-epicatechin (EC), (−)-epicatechin gallate (ECG), (−)-epigallocatechin (EGC), and (−)-epigallocatechin gallate (EGCG) were purchased from Wako Pure Chemical Industries, Ltd. (Osaka, Japan). LC-MS grade water and acetonitrile and high-performance liquid chromatography (HPLC) grade acetonitrile and methanol were purchased from Thermo Fisher Scientific Inc. (Waltham, MA, USA). Water for HPLC was purchased from Burdick & Jackson (Muskegon, MI, USA). Cellulase (100,000 units/g, EC 3.2.1.6; Lyven Co., Colombelles, France) and tannase (EC 3.1.1.20; Kikkoman, Chiba, Japan) were obtained from Bision Corp. (Seoul, Korea). Fetal bovine serum (FBS) was purchased from Hyclone (Logan, UT, USA). All other chemicals were of American Chemical Society grade or higher.

PC-12 cell line (murine adrenal gland cell; CRL-1721™), RAW 264.7 (murine macrophages; TIB-71™), and DLD-1 (human colon cancer cell; CCL-221™) were purchased from American Type Culture Collection (Manassas, VA, USA). E0771 cell line (murine breast cancer cell; #940001) was purchased from CH3 BioSystems LLC (Amherst, NY, USA).

### 2.2. Preparation of the Purified FLG and FLA from GTE

Fresh green tea leaves were harvested from May to June in 2017 (Osulloc Farm Corp., Jeju-do, Korea), and then dried. To prepare GTEs, dried green tea leaves were soaked in a 10-fold solution of 70% (*v/v*) aqueous ethanol at 60 °C with stirring for 3 h, the ethanol in the extract was removed using an evaporator (Hei-VAP; Heidolph Instruments, Schwabach, Germany), and then the extract was filtered using a 20 μm filter (Pall Corp., Port Washington, NY, USA) and solidified using a KL-8 spray dryer (Seogang Engineering, Cheonan, Korea).

**Figure 1:**
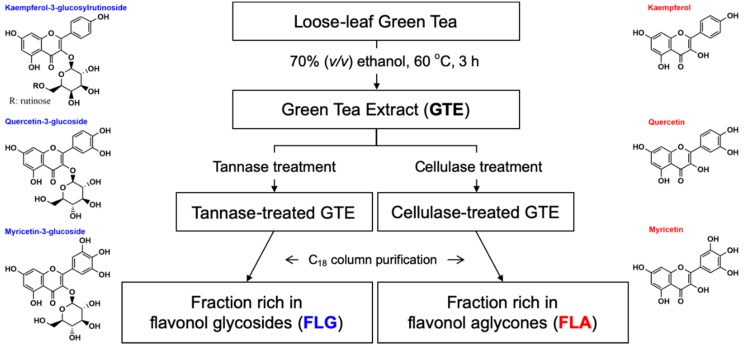
Diagram of the extraction and the purification processes with enzymatic treatment to obtain fractions rich in flavonol glycosides (FLG) and flavonol aglycones (FLA). The chemical names in blue and red indicate flavonol glycosides and flavonol aglycones, respectively.

To obtain purified FLG, GTE aqueous solution (1% (*w/v*)) of pH 5.0 was reacted with 1% (*v/v*) tannase (500 units/mL) in a thermoshaker (Eppendorf AG, Hamburg, Germany) for 14 h at 40 °C. The enzymatic reaction was ceased by heating at 90 °C for 20 min. To obtain purified FLA, similar conditions were applied using cellulase instead of tannase. These enzyme-treated GTEs were then freeze dried. The green tea extraction process with enzymatic treatment to obtain purified flavonol glycoside- and aglycone-rich fractions is shown in Figure 1.

Preparative HPLC was used to remove sugars, gallic acid, and catechins and to obtain FLG and FLA from the enzyme-treated GTEs [19]. In detail, 5 g of freeze-dried enzyme-treated GTEs were solubilized in 50 mL of 60% aqueous methanol, and then passed through a 0.45 µm polyvinylidene fluoride syringe filter (Pall Corp.) after 10 min of sonication. The filtered enzyme-treated GTEs were loaded into a preparative liquid chromatography system (ÄKTA Purifier 10; GE Healthcare, Stockholm, Sweden) coupled with a photodiode array detector at wavelengths of 254 and 365 nm. Fractionation was conducted using an octadecyl-silica (ODS) AQ-HG column (column volume (CV) = 78.5 mL, 120 Å, 10 μm, 20 × 250 mm; YMC, Kyoto, Japan). Gradient elution was performed with water (solvent A) and acetonitrile (solvent B) as follows: 0.45 CV, 84% A/16% B; 1.80 CV, 84–80% A/16–20% B; 0.45 CV, 80% A/20% B; 0.45 CV, 80–78% A/20–22% B; 0.45 CV 78–20% A/22–80% B; 0.45 CV, 20–0% A/80–100% B; 0.45 CV, 0–100% A/100–0% B; 0.45 CV, 100% A/0% B. The flow rate and injection volume were 10 mL/min and 5 mL, respectively. Fractions from each cycle were divided into two and collected in separate bottles via repeated cycles of injections. After confirming the absence of catechins in the second fraction in each cycle, the fractions were combined. The fractions were condensed using evaporators (Hei-VAP; Heidolph Instruments), dehydrated using a freeze dryer (FreeZone; Labconco Corp., Kansas City, MO, USA), and then maintained at −20 °C prior to analyses.

### 2.3. Total Phenolic and Flavonoid Content in FLG and FLA

The total phenolic content in FLG and FLA was determined using a colorimetric method with Folin–Ciocalteu’s phenol reagent [20,21]. First, 10 mg of catechin, gallic acid, FLG, or FLA were dissolved in 1 mL of DMSO, and then diluted 10 times with deionized water. Then, 200 μL each of the resulting mixture were mixed with deionized water (2.6 mL). Folin–Ciocalteu’s phenol reagent (200 μL) was added to each mixture at 0 min. Na_2_CO_3_ solution (7% (*w/v*); 2 mL) was added at 6 min. At 90 min, the absorbance was measured at 750 nm using a spectrophotometer (SPECTRONIC 200; Thermo Fisher Scientific Inc. (Waltham, MA, USA)). The total phenolic content is presented as “mg gallic acid equivalents (GAE)/g DW” of FLG or FLA.

The total flavonoid content was measured using the method reported by Kim et al. [21]. Briefly, a 500 μL portion of FLG or FLA was mixed with deionized water (3.2 mL), and then NaNO_2_ (5% (*w/v*); 150 μL) was added. AlCl_3_ (10% (*w/v*); 150 μL) was added at 5 min and NaOH (1 M; 1 mL) was added at 6 min. The absorbance was measured immediately at 510 nm using a spectrophotometer (SPECTRONIC 200). The total flavonoid content is expressed as “mg catechin equivalents (CE)/g DW” of FLG or FLA.

### 2.4. Quantification and Mass Identification of Flavonols

Dried FLG and FLA and their reaction mixtures were solubilized in 10% (*v/v*) DMSO in aqueous methanol with 20 min of sonication, and then passed through a 0.2 μm regenerated cellulose syringe filter (Sartorius Stedim Biotech GmbH, Göttingen, Germany). The filtered samples were analyzed using an Alliance HPLC apparatus (Waters, Milford, MA, USA) equipped with an auto-sampler, a quaternary pump, and a UV detector with a Poroshell 120 SB ODS column (120 Å, 2.7 μm, 4.6 × 150 mm; Agilent, Santa Clara, CA, USA). The column temperature was maintained at 30 °C. Catechins were detected at 275 nm. Flavonols and flavones were monitored at 365 nm. The injection volume was 10 μL. The flow rate was 0.8 mL/min. The mobile phases were 0.1% (*v/v*) formic acid in water (solvent C) and 0.1% (*v/v*) formic acid in acetonitrile (solvent D). All solvents used were filtered and degassed. The linear gradient was as follows: 92% C/8% D at 0 min, 92% C/8% D at 2 min, 88% C/12% D at 3 min, 84% C/16% D at 4 min, 84% C/16% D at 12 min, 80% C/20% D at 15 min, 80% C/20% D at 18 min, 76% C/24% D at 21 min, 70% C/30% D at 22 min, 70% C/30% D at 26 min, 50% C/50% D at 28 min, 50% C/50% D at 30 min, 20% C/80% D at 32 min, 20% C/80% D at 33 min, 92% C/8% D at 34 min, and 92% C/8% D at 35 min.

High-resolution mass spectrometry was conducted using a DIONEX UltiMate 3000 UHPLC apparatus (Thermo Fisher Scientific Inc. (Waltham, MA, USA)) equipped with an ACQUITY UPLC BEH ODS column (Waters; 130 Å, 1.7 μm, 2.1 × 100 mm) and a hybrid quadrupole-FT/Orbitrap mass (Q Exactive Plus System; Thermo Fisher Scientific Inc. (Waltham, MA, USA)) in the negative ion mode with heated electrospray ionization. The mobile phases were 0.1% (*v/v*) formic acid in water (solvent E) and 0.1% (*v/v*) formic acid in acetonitrile (solvent F). All solvents were filtered and degassed. The elution program was as follows: 90% E/10% F at 0 min, 90% E/10% F at 0.5 min, 50% E/50% F at 8 min, 20% E/80% F at 9 min, 100% E/0% F at 9.1 min, 100% E/0% F at 11.5 min, 90% E/10% F at 12 min, and 90% E/10% F at 15 min. The flow rate was 0.3 mL/min. The column temperature was 30 °C and the injection volume was 1 μL. Mass spectra were acquired under the following conditions: mass range of 100–1500, spray voltage was 3.5 kV, sheath gas flow rate was 45 arb, auxiliary gas flow rate was 10 arb, auxiliary gas heater temperature was 350 °C, and capillary temperature of fraction was 350 °C. Phenolic compounds were identified by metabolite profiling by referring to the literature [22,23], MS/MS spectral, and mass library searches [24].

### 2.5. Measurements of Antioxidant Capacities of FLG and FLA

#### 2.5.1. Antioxidant Capacity Measurements with ABTS Radicals

The antioxidant capacities of FLG and FLA were determined following the methods described by Kim et al. [21]. The radical solution of ABTS (980 μL) with an absorbance of 0.650 ± 0.020 at 734 nm was reacted with 20 μL of FLG or FLA at 37 °C for 10 min. The absorbance was measured at 734 nm using a spectrophotometer (SPECTRONIC 200). Antioxidant capacities are presented as “mg vitamin C equivalents (VCE)/g DW” of FLG or FLA.

#### 2.5.2. Antioxidant Capacity Measurements with DPPH Radicals

Antioxidant capacity was also evaluated following the method described by Kim et al. [21], with modifications. The absorbance of DPPH radicals was set to 0.650 ± 0.020 at 517 nm. The reaction between DPPH radicals and FLG or FLA was allowed to proceed at ambient temperature for 30 min. At 30 min, the absorbance of the resulting solution was measured at 517 nm using a spectrophotometer (SPECTRONIC 200). Antioxidant capacities are presented as “mg VCE/g DW” of FLG or FLA.

### 2.6. Measurements of Intracellular Oxidative Stress of FLG and FLA

Intracellular oxidative stress levels were evaluated using DCFH-DA following previously described methods [25]. Briefly, PC-12 cells (2 × 10^4^ cells/well) were incubated for 3 h in a humidified incubator with 5% CO_2_ at 37 °C. PC-12 cells with 50 μM DCFH-DA or 5 μM DHE in Hanks’ balanced salt solution (HBSS) were incubated for 30 min, and then treated with 100 μM H_2_O_2_ in HBSS for 1 h. Fluorescence was gauged using a microplate reader (Infinite M200; Tecan Austria GmbH, Grödig, Austria) at an excitation wavelength of 485 nm and emission wavelength of 530 nm. The intracellular oxidative stress levels are presented as the percentage (%) decrease in fluorescence intensity relative to the control (100%).

### 2.7. Anti-Inflammatory Effects of FLG and FLA

RAW 264.7 cells were maintained in DMEM with 10% FBS and 1% penicillin/streptomycin solution in a humidified incubator with 5% CO_2_ at 37 °C. RAW 264.7 cells were pre-treated with FLG and FLA for 2 h, and then treated with 10 ng/mL LPS for 6 h. After incubation, RAW 264.7 cells were washed two times with PBS. RNA was isolated using TRIzol reagent (Thermo Fisher Scientific Inc. (Waltham, MA, USA)), according to the manufacturer’s protocol. Equal amounts (1 μg each) of RNA were used to synthesize cDNA using the RevertAid First Strand cDNA Synthesis Kit (Thermo Fisher Scientific Inc. (Waltham, MA, USA)) following the manufacturer’s instructions. Relative levels of mRNA were measured using the quantitative real-time polymerase chain reaction (qPCR; refer to the method in the Supplementary Materials and Table S1) system (CFX-96; Bio-Rad Laboratories, Hercules, CA, USA) with appropriate primers (Table S2, Supplementary Materials).

### 2.8. Anticancer Effects of FLG and FLA

DLD-1 cells were cultured in DMEM supplemented with 10% FBS and 0.1% penicillin/streptomycin solution in a humidified incubator at 37 °C with 5% CO_2_. E0771 cells were grown in RPMI-1640 supplemented with 10% FBS and 0.1% penicillin/streptomycin and were cultured under the same temperature and humidity conditions. Cell viability was confirmed based on the formation of a purple formazan metabolite from MTT, which was solubilized with DMSO. DLD-1 and E0771 cells in 96-well plates (5 × 10^3^ cells/well) were incubated with samples for 24 h with or without paclitaxel (10 nM), and then MTT (1 mg/mL) was added and the cells were incubated for 2 h. Purple formazan crystals were dissolved in DMSO and absorbance was measured at a wavelength of 570 nm using a microplate reader (Infinite M200 Pro; Tecan, Männedorf, Switzerland). The results are represented as the percent absorbance relative to that of the control cells.

### 2.9. Statistical Analyses

Data are expressed as means ± standard error of the mean (*n* = 3). One-way analysis of variance and Tukey–Kramer honestly significant difference (HSD) test with *p* < 0.05 were implemented in JMP 12 for Windows 7 or higher (SAS Institute Inc., Cary, NC, USA). Statistical analyses of the qPCR results were performed using SPSS 22.0 (SPSS Inc., Chicago, IL, USA).

## 3. Results

### 3.1. Phenolic Compositions of FLG and FLA

**Figure 2:**
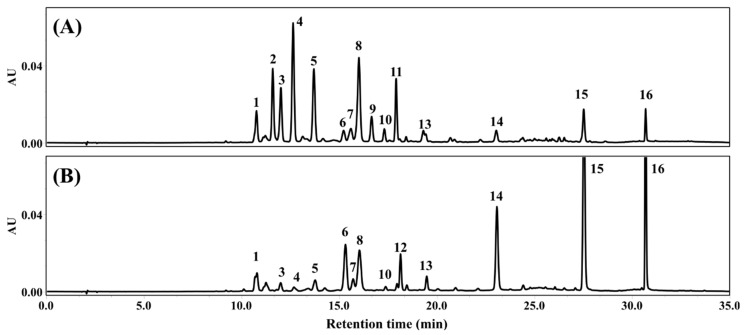
HPLC chromatograms of the fractions rich in (**A**) flavonol glycosides (FLG) and (**B**) flavonol aglycones (FLA) at 365 nm. Refer to Table 1 for identification of each numbered peak of the 16 phenolics.

The chromatographic profiles of FLG and FLA are shown in Figure 2. Each peak for FLG and FLA was tentatively assigned according to the flavonol and flavone backbones and their sugar moieties by tandem LC-MS/MS (Table 1**.**). The flavonoids detected in FLG obtained from aqueous GTE after treatment with tannase included two apigenin derivatives (apigenin-6-*C*-glucosyl-8-*C*-arabinoside and apigenin-6-*C*-glucoside (or an isomer of apigenin-6-*C*-glucoside)), kaempferol and three of its derivatives (kaempferol-3-*O*-glucosylrutinoside, kaempferol-3-*O*-rhamnosylgalactoside, and kaempferol-3-*O*-rhamnosylglucoside), myricetin and two of its derivatives (myricetin-3-*O*-galactoside and myricetin-3-*O*-glucoside), and quercetin and six of its derivatives (quercetin-3-*O*-galactosylrutinoside, quercetin-3-*O*-glucosylrutinoside, quercetin-3-*O*-rhamnosylgalactoside, quercetin-3-*O*-rhamnosylglucoside, quercetin-3-*O*-galactoside, and quercetin-3-*O*-glucoside) (Table 1). FLA contained the same flavonoids except myricetin-3-*O*-galactoside, quercetin-3-*O*-galactoside, and kaempferol-3-*O*-glucosylrutinoside (Table 1).

The 16 flavonoids found in the extracts were identified as aglycones and glycosides of flavones and flavonols (Figure 2 and Table 1). The glycosides in FLG and FLA were 3-*O*-glycosyl flavonols and 6-*C*-glycosyl flavones. The total amount of the three flavonol aglycones myricetin, quercetin, and kaempferol (88.86 mg/g) in FLA was five times higher than that in FLG (17.78 mg/g) (Table 2), indicating that the glycosides (peaks 2, 3, 4, 5, 9, and 11) in green tea are hydrolyzed by cellulase treatment to yield their corresponding aglycones (peaks 14, 15, and 16) (Figure 2). The detachment of sugars from some flavonoids such as myricetin-3-*O*-galactoside (peak 2), myricetin-3-*O*-glucoside (peak 3), quercetin-3-*O*-galactosylrutinoside (peak 4), quercetin-3-*O*-glucosylrutinoside (peak 5), and kaempferol-3-*O*-glucosylrutinoside (peak 11) resulted in increased amounts of their corresponding aglycones in FLA (Figure 2 and Table 2).

Catechins such as EC, ECG, EGC, and EGCG are a major group of phenolics in green tea. However, catechins were not detected in FLG and FLA in this study as they were eliminated during preparative purification (Table 2). FLA contained higher amounts of quercetin-3-*O*-rhamnosylgalactoside (peak 6) and kaempferol-3-*O*-rhamnosylrustinoside (peak 12) than those in FLG (data not shown). Due to the different enzyme treatments, there were significant (*p* < 0.05) differences in the total flavonoid and phenolic content of FLG and FLA. The total phenolic content in FLG and FLA was 401.48 and 568.89 mg GAE/g DW, respectively (Table 2). The content of total phenolics and total flavonoids in FLA were approximately 42% and 32% higher than those in FLG, respectively (Table 2).

### 3.2. Antioxidant Capacities of FLG and FLA

The antioxidant capacities of GTE were 1637.06 ± 16.67 and 1335.27 ± 36.35 mg VCE/g DW of GTE, as measured using the ABTS and DPPH assays, respectively (Table 3). The antioxidant capacities of FLA and FLG measured using ABTS radicals decreased to approximately 53.2–83.1% compared with those of GTE, whereas FLA and FLG had approximately 35.2% and 61.2% lower DPPH radical scavenging antioxidant capacity than that of GTE, respectively (Table 3). The results of the ABTS and DPPH assays for tannase-treated GTE (crude extract with no purification) were higher (i.e., 2011 and 1317 mg VCE/g DW, respectively) than those for GTE due to extra gallic acid (data not shown).

### 3.3. Effects of FLG and FLA on Intracellular Oxidative Stress

**Figure 3:**
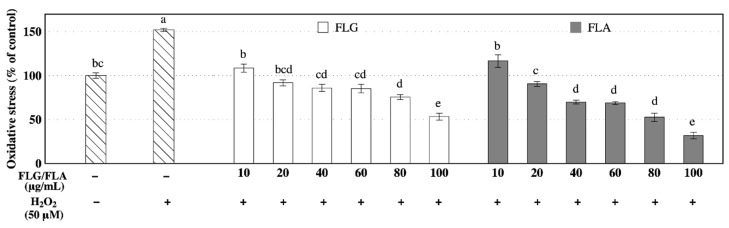
Effects of the fractions rich in flavonol glycosides (FLG) and flavonol aglycones (FLA) on intracellular oxidative stress in neuron-like PC-12 cells. Lowercase letters on the bars indicate significant differences according to the Tukey–Kramer HSD test (*p* < 0.05).

Intracellular oxidative stress levels in neuron-like PC-12 cells are summarized in Figure 3. Reactive oxygen species (ROS) levels in PC-12 cells was increased to 150% by oxidative stress (50 μM H_2_O_2_) compared with those in control cells (100%). Pretreatment of PC-12 cells with FLG and FLA decreased the levels of intracellular oxidative stress induced by 50 μM H_2_O_2_ in a dose-dependent manner. At concentrations of 10 and 20 μg/mL, PC-12 cells treated with FLG and FLA showed no significant (*p* < 0.05) difference in the levels of intracellular oxidative stress compared with those of the control.

### 3.4. Anti-Inflammatory Effects of FLG and FLA

**Figure 4:**
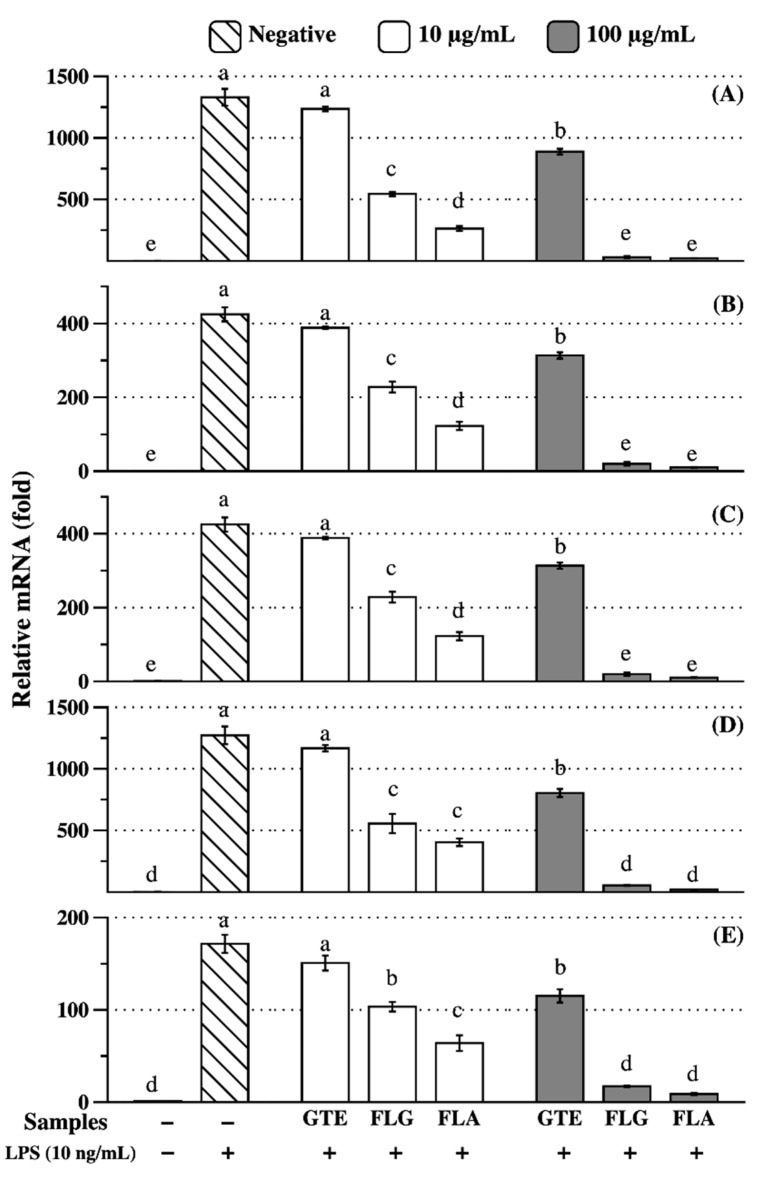
Anti-inflammatory effects of green tea extract (GTE), the fraction rich in flavonol glycosides (FLG), and the fraction rich in flavonol aglycones (FLA) on inflammatory factors, including (**A**) IL-1β, (**B**) IL-6, (**C**) iNOS, (**D**) COX-2, and (**E**) MMP9 in RAW 264.7 cells. “+” indicates that LPS was applied before sample treatment. Lowercase letters on the bars indicate significant differences according to the Tukey–Kramer HSD test (*p* < 0.05).

Anti-inflammatory effects of GTE, FLG, and FLA were investigated in RAW 264.7 cells. Genes encoding pro-inflammatory markers, such as cyclooxygenase-2 (COX-2), inducible nitric oxide synthase (iNOS), interleukin-1β (IL-1β), IL-6, and matrix metalloproteinase 9 (MMP9), were highly expressed in RAW 264.7 cells treated with LPS (Figure 4). GTE at 10 µg/mL concentration did not significantly (*p* < 0.05) reduce the expression of pro-inflammatory genes, whereas 100 µg/mL GTE significantly (*p* < 0.05) reduced pro-inflammatory gene expression (Figure 4). However, FLG and FLA decreased mRNA expression of pro-inflammatory genes in a dose-dependent manner (Figure 4). RAW 264.7 cells treated with FLG and FLA at 100 µg/mL concentration had similar levels of inflammation-related gene expression to those of control cells. However, no significant differences were found in the effects on lipid metabolism (Figure S1, Supplementary Materials)

### 3.5. Anticancer Effects of FLG and FLA

**Figure 5:**
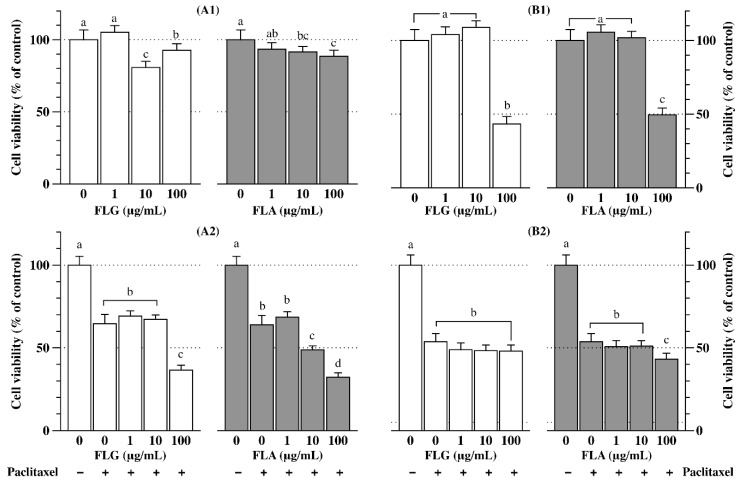
Anticancer effects of the fractions rich in flavonol glycosides (FLG) and in flavonol aglycones (FLA). (**A1**) DLD-1 cells treated with samples for 24 h, (**A2**) DLD-1 cells treated with samples and paclitaxel (10 nM) for 24 h, (**B1**) E0771 cells treated with samples for 24 h, (**B2**) E0771 cells treated with samples and paclitaxel (10 nM) for 24 h. Different letters on the bars indicate significant differences according to the Tukey–Kramer HSD test (*p* < 0.05).

Two adenoma cell lines, namely DLD-1 and E0771, were used to examine the anticancer effects of FLG and FLA with or without a chemotherapeutic agent (paclitaxel). At 10 and 100 µg/mL concentrations, FLG and FLA significantly (*p* < 0.05) reduced DLD-1 cell viability compared with that of the control (100%) (Figure 5A1). Co-treatment with the anticancer drug paclitaxel (10 nM) decreased the viability of DLD-1 cells to 64% compared with that of the control (Figure 5A2). The viability of DLD-1 cells upon co-treatment with paclitaxel and FLG (100 µg/mL) was approximately 36% of that of the control cells. Co-treatment with paclitaxel and FLA at 10 and 100 µg/mL concentrations resulted in decreases in the viability of DLD-1 cells to 49% and 32%, respectively, compared with that of the control, indicating that FLA and paclitaxel have synergistic effects on cancer cell death (Figure 5A2).

Similarly, treatment with FLG or FLA at 100 µg/mL concentration decreased the viability of the breast cancer cell line E0771 (Figure 5B1). However, co-treatment with paclitaxel and FLG or FLA did not significantly alter E0771 cell viability compared with that of the control treated with paclitaxel only (Figure 5B2), suggesting that there are no synergistic anticancer effects of FLG or FLA and the drug on E0771 cells.

## 4. Discussion

Green tea contains a variety of polyphenols; in particular, monomeric flavonols, that is, flavan-3-ols (catechins), are dominant compounds in GTE. The total phenolic and flavonoid content in GTE varies according to the solvent [26]. The estimated flavonol glycoside content in green teas is 2.4–5.1 mg/g of dried tea [27]. The total polyphenol content is approximately 30% of dried tea, whereas flavonols account for only 0.4% of dried tea [2,3]. Using over 40% (*v/v*) aqueous ethanol as a solvent for extraction, approximately 30% (*w/w*) catechins can be extracted; the portion of catechins in the extract increases as the extraction time increases [28].

Senanayake [29] previously reported that green tea polyphenols exert antioxidative effects via various mechanisms of action. The antioxidative effects of green tea are primarily attributed to monomeric flavan-3-ols; however, information on other compounds, such as flavonols, is limited. Plumb et al. [11] found that the antioxidative effect of flavonol glycosides gradually decreases as the glycoside length increases (e.g., from monoglycosides to triglycosides). Ratty and Das [30] reported that flavonol glycosides have lower antiperoxidative activity than the corresponding aglycones owing to the masking effect of complex glycoside structures. Heim et al. [31] pointed out that the decreased antioxidant capacities could be explained as follows: (i) the free 3-OH in the C-ring of flavonoids is crucial for maintaining antioxidant capacity, (ii) coplanarity of the B-ring of flavonoids is destroyed by the attachment of the glycoside to the 3-OH position, and (iii) the hydrophilicity of glycosides may alter the molecular accessibility during radical scavenging.

Similar to the crude extract results of this study, it has been reported that tannase treatment increased the antioxidant capacity of green tea [32]. The antioxidant capacity of kaempferol glycosides decreased to 32–39% [11]. In this context, FLG had a low antioxidant capacity due to its glycosylated structure, whereas FLA recovered the antioxidant capacity by obtaining free -OH from the enzymatic treatment. FLA has higher total phenolic and flavonoid content than that of FLG (Table 2). FLA contained approximately 400% higher aglycone content (kaempferol, quercetin, and myricetin) than that of FLG (Table 2). Therefore, FLA showed a higher antioxidant capacity than that of FLG (Table 3).

Many studies have reported the antioxidative effects of single flavonoid compounds [21,30,31,33]. Kaempferol and quercetin found in FLA in this study have been reported to show higher antioxidant capacities than vitamin C [33]. These flavonoids attenuate oxidative stress in cells, partly by scavenging ROS [25]. Consistent with these previous findings, FLG and FLA in green tea attenuated the level of intracellular oxidative stress in a dose-dependent manner (Figure 3). FLA showed a greater reduction in intracellular oxidative stress at concentrations of above 40 μg/mL than FLG (Figure 3). The reduction in intracellular oxidative stress in PC-12 cells can possibly be associated with antioxidative polyphenols, such as myricetin, quercetin, kaempferol, and their glycosides found in FLG and FLA.

Catechins are unstable in aqueous and digestive conditions, resulting in epimerization or ring fission depending on various factors, such as moisture, pH, and microbiota [34,35,36,37]. The bioavailability of plant polyphenols has important nutritional implications; small quantities are generally available in the plasma after ingestion. Numerous studies have attempted to improve the bioavailability of catechins by changing their physical properties, altering acidity, and carrying out co-treatment with inhibitors of efflux transporters [28,38,39,40]. An interaction between catechins and the other compounds in GTE has not been demonstrated. A recent study has shown that green tea flavonols enhance the bioavailability of catechins in a Caco-2 cell model, partly due to the antioxidative effects of flavonols [19]. In this study, we evaluated the role of green tea flavonols by summing their functional potency, as the antioxidative effects have already been evaluated. Flavonols in the form of glycosides are more stable than those in the form of aglycones under digestive conditions, implying that flavonol glycosides may be more stable than monomeric catechins in green tea [41,42]. Although bioactive components in green tea interact with each other and increase, decrease, or preserve the potency of the physiological effects of other components, flavonols in green tea may play an important role as functional food materials.

Inflammation contributes to defense against exogenous, harmful stimuli [14]. Extracellular pathogenic agents, such as LPS, bind to toll-like receptor 4 (TLR4) to initiate an innate immune response [43]. Numerous immune cells migrate to the inflamed site to eliminate pathogens and damaged cells. As the immune response is an important process for cell survival, it must be tightly regulated to maintain health. Interestingly, TLR4 also recognizes free fatty acids as an agonist [44]. Therefore, non-esterified fatty acids can provoke an inflammatory response even in the absence of extracellular stimulants, and this is referred to as chronic inflammation [43]. As the inflammatory response is known to interfere with insulin signaling, chronic inflammation is regarded as a causal factor for the progression of various metabolic disorders, such as obesity, insulin resistance, hyperlipidemia, hyperglycemia, type 2 diabetes, certain types of cancer, and atherosclerosis [43,45]. Therefore, the appropriate modulation of inflammatory response is important to maintain health.

The green tea-derived flavonol-rich fractions FLG and FLA used in this study exhibited anti-inflammatory effects. The inhibitory effects of flavonols, regardless of the presence of glycosylated branches, on inflammation were much greater than those of GTE (Figure 4). Considering the catechin content in GTE (approximately 35% (*w/w*); data not shown) and the flavonol content in FLG (1.8% (*w/w*)) and FLA (8.9% (*w/w*)) detected in this study (Table 2), it is reasonable to conclude that the anti-inflammatory effects of flavonols are greater than those of catechins. The anti-inflammatory effects of FLA were greater than those of FLG. As the major difference between FLG and FLA is the presence or absence of glycosylation on the flavonol backbone, we can assume that enzyme-mediated deglycosylation would affect the bioavailability and efficiency of flavonols in the attenuation of pro-inflammatory responses. Owing to their suppressive effects on inflammation-related genes in macrophages, green tea-derived flavonols are potential novel therapeutic agents for inflammatory disorders.

During inflammation, a large amount of chemokines is released to facilitate immune cell migration [46]. Similarly, cancer cells require chemokines for invasion and metastasis [47,48]. Therefore, the inflammatory response is also closely associated with the development of cancer, and flavonols with anti-inflammatory effects are also expected to exert anticancer effects [49].

FLA treatment had no cytotoxic effects on colon cancer cells (Figure 5A1). Co-treatment with FLA (10 and 100 µg/mL) and paclitaxel resulted in the synergistic inhibition of colon cancer cell growth (Figure 5A2). FLG at 10 and 100 µg/mL concentrations significantly inhibited the growth of colon cancer cells (Figure 5A1). Furthermore, co-treatment with FLG (100 µg/mL) and paclitaxel resulted in the synergistic inhibition of colon cancer cell growth. Consistent with our results, co-treatment with EGCG and paclitaxel significantly reduced the growth of breast cancer cells compared with that of EGCG or paclitaxel alone [50]. EGCG, a component of GTE, in combination with paclitaxel shows anticancer effects [51]. FLG and FLA at a concentration of 100 µg/mL decreased the viability of breast cancer cells in our study (Figure 5B1). However, breast cancer cell growth was only inhibited in response to co-treatment with FLA (100 µg/mL) and paclitaxel (Figure 5B2). The results of this study (Figure 5) suggest that colorectal cancer and breast cancer cells have different sensitivities to paclitaxel.

## 5. Conclusions

Using HPLC-MS analysis, we determined that FLA contained higher concentrations of flavonoid aglycones than that of FLG. In this study, 16 derivatives of apigenin, kaempferol, myricetin, and quercetin were identified in FLG and 13 in FLA. We concluded that FLG and FLA without catechins from green tea have potent antioxidant capacities and reduce oxidative stress in PC-12 cells. FLG and FLA significantly reduced the mRNA expression of inflammation-related genes in murine RAW 264.7 macrophages. Additionally, the growth of DLD-1 and E0771 cancer cells was synergistically inhibited by co-treatment with FLG or FLA and paclitaxel. Taken together, these findings indicate that FLG and FLA from green tea have beneficial effects on health, partly due to their antioxidant components and anti-inflammatory and anticancer activities. Further studies are needed to investigate the effects of FLG and FLA on inflammation and cancer using in vivo animal models.

## Figures and Tables

**Table 1:** Identification of individual phenolics in the fractions rich in flavonol glycosides and flavonol aglycones from green tea extract.

Peak No.	Molecular ion (*m/z*, [M-H]^−^)	Formula	Fragmentation	Identification ^a^
1	563.14020	C_32_H_27_O_14_	545, 473, 443, 383, 353	Apigenin-6-*C*-glucosyl-8-*C*-arabinoside
2	479.08228	C_21_H_19_O_13_	316	Myricetin-3-*O*-galactoside
3	479.08267	C_21_H_19_O_13_	316	Myricetin-3-*O*-glucoside
4	771.19812	C_33_H_39_O_21_	301	Quercetin-3-*O*-galactosylrutinoside
5	771.19812	C_33_H_39_O_21_	301	Quercetin-3-*O*-glucosylrutinoside
6	609.14581	C_27_H_29_O_16_	301	Quercetin-3-*O*-rhamnosylgalactoside
7	609.14606	C_27_H_29_O_16_	301	Quercetin-3-*O*-rhamnosylglucoside
8	431.09787	C_26_H_19_O_10_	353, 269	Apigenin-6-*C*-glucoside or isomer
9	463.08801	C_21_H_19_O_12_	301	Quercetin-3-*O*-galactoside
10	463.08829	C_21_H_19_O_12_	301	Quercetin-3-*O*-glucoside
11	755.20367	C_33_H_39_O_20_	285	Kaempferol-3-*O*-glucosylrutinoside
12	593.15009	C_27_H_29_O_15_	285	Kaempferol-3-*O*-rhamnosylgalactoside
13	593.15131	C_27_H_29_O_15_	285	Kaempferol-3-*O*-rhamnosylglucoside
14	317.02951	C_15_H_9_O_8_		Myricetin
15	301.03491	C_15_H_9_O_7_		Quercetin
16	285.03983	C_15_H_9_O_6_		Kaempferol

^a^ Identification inferred from the literature except myricetin, quercetin, and kaempferol.

**Table 2:** Phenolic concentrations of the fractions rich in flavonol glycosides (FLG) and flavonol aglycones (FLA).

	Myricetin ^a^	Quercetin ^a^	Kaempferol ^a^	Epicatechins ^ab^	Total Flavonoids ^c^	Total Phenolics ^d^
FLG ^e^	4.15 ± 0.16 ^Bf^	7.72 ± 0.43 ^B^	5.91 ± 0.24 ^B^	N/D ^g^	132.76 ± 0.98 ^B^	401.48 ± 1.52 ^B^
FLA ^h^	15.94 ± 0.48 ^A^	38.21 ± 1.20 ^A^	34.71 ± 1.19 ^A^	N/D	174.67 ± 1.33 ^A^	568.89 ± 1.99 ^A^

^a^ Concentrations are expressed as mg/g sample. ^b^ No catechins were detected due to purification through a preparatory ODS column. (−)-Epicatechins included (−)-epigallocatechin, (−)-epigallocatechin gallate, (−)-epicatechin, and (−)-epicatechin gallate. ^c^ Concentrations are expressed as mg catechin equivalents/g DW. ^d^ Concentrations are expressed as mg gallic acid equivalents/g DW. ^e^ FLG, fraction rich in flavonol glycosides. ^f^ Results are shown as means ± standard error of the mean (*n* = 3). ^g^ N/D, not detected. ^h^ FLA, fraction rich in flavonol aglycones. Different superscripted uppercase letters in the same column indicate significant differences (*p* < 0.05) of the means.

**Table 3:** Antioxidant capacities of green tea extract (GTE), the fraction rich in flavonol glycosides (FLG), and the fraction rich in flavonol aglycones (FLA).

	Antioxidant Capacities (mg VCE/g DW)	
	ABTS	DPPH
GTE	1637.06 ± 16.67 ^Aa^	1335.27 ± 36.35 ^A^
FLG	870.20 ± 18.60 ^C^	518.25 ± 32.02 ^C^
FLA	1360.80 ± 8.25 ^B^	864.60 ± 19.35 ^B^

^a^ Different superscripted uppercase letters in the same column indicate significant differences according to the Tukey–Kramer HSD test (*p* < 0.05).

## Supplementary Materials

The following are available online at https://www.mdpi.com/2076-3921/8/8/278/s1. The qPCR protocol; Table S1, qPCR mix and reaction condition; Table S2, Sequences of qPCR primers; Figure S1, Modulating effects on lipid metabolism-related genes in 3T3-L1.

## Abbreviations

AAPH	2,2′-azobis(2-amidinopropane) dihydrochloride
ABTS	2,2′-azino-bis(3-ethylbenzothiazoline-6-sulfonic acid) diammonium salt
CE COX-2 CV DCFH-DA	catechin equivalents cyclooxygenase-2 column volume 2′,7′-dichlorofluorescein diacetate
DHE	dihydroethidium
DMSO	dimethyl sulfoxide
DPPH	1,1-diphenyl-2-picrylhydrazyl
DMEM	Dulbecco’s modified Eagle’s medium
DW	dry weight
EC	(−)-epicatechin
ECG	(−)-epicatechin gallate
EGC	(−)-epigallocatechin
EGCG	(−)-epigallocatechin gallate
FBS	fetal bovine serum
FLA	fraction rich in flavonol aglycones
FLG	fraction rich in flavonol glycosides
GAE GTE	gallic acid equivalents green tea extract
HPLC	high-performance liquid chromatography
HBSS HSD	Hanks’ balanced salt solution honestly significant difference
IL	interleukin
iNOS	inducible nitric oxide synthase
LPS MMP9	Lipopolysaccharide matrix metalloproteinase 9
MTT	3-(4,5-dimethylthiazol-2yl)-2,5-diphenyltetrazolium bromide
ODS PBS	octadecyl-silica phosphate-buffered saline
qPCR	quantitative real-time polymerase chain reaction
ROS	reactive oxygen species
RPMI	Roswell Park Memorial Institute
TLR4	toll-like receptor 4
VCE	vitamin C equivalents
