# Supplementary material for: Antioxidative, Anti-Inflammatory, and Anticancer Effects of Purified Flavonol Glycosides and Aglycones in Green Tea
Source: Antioxidants (Basel). 2019 Aug 5;8(8):278. doi: 10.3390/antiox8080278 (PMC6719943; doi:10.3390/antiox8080278)
Supplement: Supplementary file 1 [file antioxidants-08-00278-s001.pdf]

Supplementary Materials

Antioxidative, Anti-inflammatory, and Anticancer Effects of Purified Flavonol Glycosides and Aglycones in Green Tea

Chan-Su Rha [1], Hyun Woo Jeong [1], Saitbyul Park [2], Siyoung Lee [3], Young Sung Jung [4], and Dae-Ok Kim [4,*]

[1] Vitalbeautie Research Division, Amorepacific Corporation R&D Center, Yongin 17074, Republic of Korea
[2] Safety and Regulatory Division, Amorepacific Corporation R&D Center, Yongin 17074, Republic of Korea
[3] Precision Medicine Research Center, Advanced Institutes of Convergence Technology, Suwon 16229, Republic of Korea
[4] Department of Food Science and Biotechnology, Kyung Hee University, Yongin 17104, Republic of Korea
* Correspondence: DOKIM05@khu.ac.kr; Tel.: +82-31-201-3796

qPCR protocol

To perform qPCR assay, synthesized cDNAs (3 μL) were mixed with Quantispeed SYBR NO-ROX premix solution (10 μL; PhileKorea, Seoul, Republic of Korea), appropriate qPCR primers (2 μL; additional information is shown in Table S1) and distilled water (5 μL). PCR assay was conducted as follows;

Table S1. qPCR mix and reaction condition.

Step	Temperature	Time	Comment
Initialize	95 °C	2 min	
Denature	95 °C	20 s	
Primer annealing / extension	60 °C	15 s	× 40 cycles
Melt Curve	60 °C–95 C°	5 s	ΔT 0.5 °C

Table S2. Sequences of qPCR primers.

Gene	Forward	Reverse
Cyclophilin	CAGACGCCACTGTCGCTTT	TGTCTTTGGAACTTTGTCTGCAA
IL-1β	TGCAGAGTTCCCCAACTGGTACATC	GTGCTGCCTAATGTCCCCTTGAATC
IL-6	AGTTGCCTTCTTGGGACTGA	TCCACGATTTCCCAGAGAAC
iNOS	AATCTTGGAGCGAGTTGTGG	CAGGAAGTAGGTGAGGGCTTG
COX-2	AGAAGGAAATGGCTGCAGAA	GCTCGGCTTCCAGTATTGAG
MMP9	CGACGACGACGAGTTGTG	CATGGGGCACCATTTGAGTT

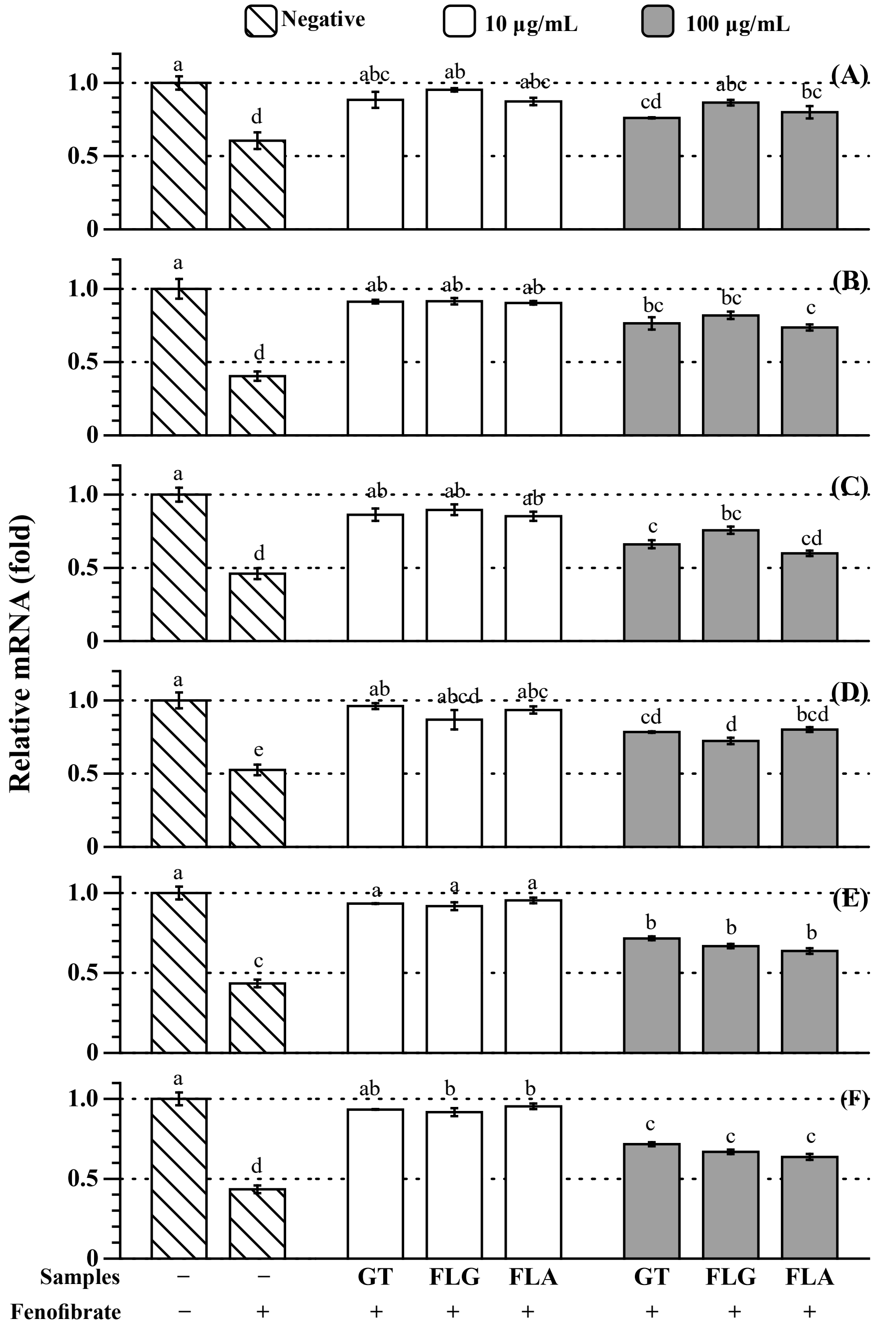

Figure S1. Modulating effects on lipid metabolism related genes in 3T3-L1. Genes: (**A**), SREBP1c (sterol regulatory element-binding proteins 1c); (**B**), ACC (acetyl-CoA carboxylase); (**C**), FAS (fatty acid synthase); (**D**), SCD1 (stearyl-CoA dehydrogenase 1); (**E**) aP2 (adipocyte protein 2); (**F**), CD36 (cluster of differentiation 36). Different letters on the bars indicate significant differences according to the Tukey–Kramer Honestly Significant Difference test ($p < 0.05$).
